# Anticonvulsant activity of aza-Biginelli derivatives related to JM-II-43A and HSAB-based rationalization of their pharmacological profile

**DOI:** 10.1007/s10822-026-00844-z

**Published:** 2026-06-12

**Authors:** Hulme Ríos-Guerra, Harold Alexis Prada-Ramírez, Benjamín Velasco-Bejarano, Margarita López-Martínez, Judith Espinosa Espinosa-Raya, Alfredo Briones-Arandas, René Miranda-Ruvalcaba, María Inés Nicolás-Vázquez, Raquel Gómez-Pliego

**Affiliations:** 1 Laboratorio de Química Multicomponente, Sección de Química Orgánica, Departamento de Ciencias Químicas, Facultad de Estudios Superiores Cuautitlán, Campo 1, Universidad Nacional Autónoma de México, Av. 1ero de Mayo S/N, Santa María Guadalupe las Torres, C.P. 54740 Cuautitlán Izcalli, Estado de México México; 2 Universidad de los Andes, Bogotá Cundinamarca, Colombia; 3 Laboratorio de Química Medicinal Verde, Sección de Química Orgánica, Departamento de Ciencias Químicas, Facultad de Estudios Superiores Cuautitlán, Campo 1, Universidad Nacional Autónoma de México, Av. 1ero de Mayo S/N, Santa María Guadalupe las Torres, C.P. 54740 Cuautitlán Izcalli, Estado de México México; 4 Departamento de Fisiología y Desarrollo Celular, Instituto Nacional de Perinatología, Montes Urales 800, Col. Lomas de Virreyes, C.P. 11000 Miguel Hidalgo, Ciudad de México, México; 5 Laboratorio Multidisciplinario en Ciencias Biomédicas, Escuela Superior de Medicina, Instituto Politécnico Nacional, Plan de San Luis y Díaz Mirón S/N, C.P. 11340 Miguel Hidalgo, Ciudad de México, México; 6 Laboratorio de Farmacología, Facultad de Medicina Humana, Universidad Autónoma de Chiapas, Décima Sur, esquina con calle Central S/N, C.P. 29000 Tuxtla Gutiérrez, Chiapas México; 7 Laboratorio de Investigación en Química Orgánica-Verde, Sección de Química Orgánica, Departamento de Ciencias Químicas, Facultad de Estudios Superiores Cuautitlán, Campo 1, Universidad Nacional Autónoma de México, Av. 1ero de Mayo s/n, Santa María Guadalupe las Torres, C.P. 54740, Cuautitlán Izcalli, Estado de México México; 8 Laboratorio de Química Computacional, Sección de Química Inorgánica, Departamento de Ciencias Químicas, Facultad de Estudios Superiores Cuautitlán, Campo 1, Universidad Nacional Autónoma de México, Av. 1ero de Mayo S/N, Santa María Guadalupe las Torres, 54740 Cuautitlán Izcalli, Estado de México México; 9 Laboratorio de Microbiología Industrial, Sección de Ciencias de la Salud Humana, Departamento de Ciencias Biológicas, Facultad de Estudios Superiores Cuautitlán, Campo 1, Universidad Nacional Autónoma de México, Av. 1ero de Mayo S/N, Col. Santa María Guadalupe las Torres, C.P. 54740 Cuautitlán Izcalli, Estado de México México

**Keywords:** Anticonvulsant activity, Aza-Biginelli products, HSAB parameters, Pentylenetetrazole model, Quantum mechanical method

## Abstract

Among the nitrogen-containing heterocyclic compounds proposed as selective modulators of γ-aminobutyric acid type A (GABA_A_) receptor subtype, dihydropyrimidinone JM-II-43A figures as a promising probe molecule for the study of neurological disorders. In this work, its anticonvulsant activity was examined in vivo, in addition to its HSAB parameters as a prospective biological predictor to compared them with its racemic aza-Biginelli homologous (**11a**–**i**) containing π − donor, σ-acceptor and σ-neutral substituents as key modulators of the physicochemical parameters. To rationalize structure–property relationships and predict trends in global chemical reactivity, their paramount global chemical reactivity descriptors (E_LUMO_, E_HOMO_, ΔE_(LUMO–HOMO)_, IP, EA, η, χ, σ, μ, and ω) were determined using quantum chemical calculations performed at DFT B3LYP/6–311 +  + G (**) levels of theory. Additionally, its anticonvulsant activity was assessed in CF1 male mice using PTZ pharmacological model. Overall, the aza-Biginelli compounds **11a** (**f**) exhibited the most pronounced seizure-inhibitory response at 10 mg/kg, outperforming the reference compound JM-II-43A at this dose. Although the overall anticonvulsant potency was moderate, all aza-Biginelli products tested showed a favorable safety profile (LD50 > 1000 mg/kg).

**Supplementary Information:**

The online version contains supplementary material available at 10.1007/s10822-026-00844-z.

## Introduction

**Fig. 1:**
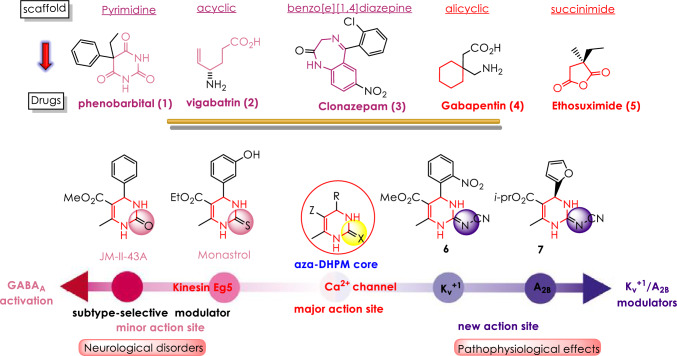
Drugs featuring pyrimidine scaffold, acyclic skeleton, benzo[*e*][1,4]diazepine, alicyclic, and succinimide are well-known antiepileptic drugs for their main action on GABA_A_ receptors (**1**–**3**) and Ca^2+^ channels (**4**–**5**). [11, 12] In this regard, aza-DHPM JM-II-43A and monastrol, originally identified as a specific inhibitor of kinesin Eg5, [13, 14] are members of a new generation of dihydropyrimidinones (thione) ligands that modulate GABA_A_ receptor. Their subtle structural effects have been found to play a crucial role in shifting affinity toward alternatives therapeutic targets (**6**–**7**)

Following the discovery of the compound Aza-Biginelli JM-II-43A (methyl 6-methyl-2-oxo-4-phenyl-3,4-dihydro-1*H*-pyrimidine-5-carboxylate) as a selective in vitro modulator of gamma-aminobutyric acid type-A (GABA_A_) receptor subtypes, specifically wild-type R1β2δ and mutated R1β2δ(E177A) receptors, [1] this privileged structure has been considered pivotal for the study of structure-structure interaction (scaffold hopping) to validate novel therapeutic applications with profound neurological implication for the treatment of diseases as epilepsy, schizophrenia, Alzheimer, and Parkinson. [2–7] Advances in this field have diversified the pursuit for new drugs chemotypes or drug candidates of this class for neurological disorders targeting to GABA_A_ receptors and Ca^2+^ cannels, [8] as well as for pathophysiological diseases associated to small molecules modulators of K_v_^+1^ channels, A_2B_ adenosine receptors [9] and cytotoxic activities (Fig. 1) [10].

In pursuit of a further insight of the neuroprotective affect exerted by compound JM-II-43A, a Monastrol-like scaffold chemotype based on the 3,4-dihydropyrimidin-2(1*H*)-one skeleton, special attention has been paid to its physicochemical features, in particular, to the formation of drug-receptor complexes, due to its inherent ability to form non-covalent bonds through its N1/C2 = O/N3 donor–acceptor-donor interaction sites, as well as to the scaffold´s propensity for π-π stacking, [15, 16]and to the selectivity of covalently bonded adduct formation between chemical xenobiotics and biological targets (drug-target interactions). Following the categorization of these sites in hard and soft nucleophiles and electrophiles from Pearson's theory of Hard and Soft, Acids and Bases (HSAB), and interpreted quantitatively in light of the Salem-Klopman equation, a significant degree of bonding selectivity between the chemical ligands and the biological target has been successfully predicted. From these fundamental chemical theories, it has been stated that the frontier orbitals (HOMO_nucleophile_/LUMO_electrophile_) of hard nucleophiles react faster with hard electrophile, and those of soft nucleophile with soft electrophile, which supports the hypothesis that electrophiles or nucleophiles ligands react preferentially with biological targets of similar hardness or softness (affinity binding). [17, 18] Therefore, integrating the conceptual principles of FO into pharmaceutical research offers a rational strategy for understanding ligand diversity-driven modulation of biological activity.

In an attempt to bridge this information gap between the strength of nucleophilic sites on the aza-Biginelli scaffold and anticonvulsant activity, in this work we endeavored to examine the relevant HSAB descriptors (σ, η and ω) for the model compound JM-II-43A, including Monastrol, and a set of its structural homologous bearing π donor, σ acceptor and σ neutral substituents in the *para*-phenyl moiety, from density functional theory. [19] The biological relevance of these quantum chemical parameters was evidenced in corroborative in vivo studies in mice using the pentylenetetrazol (PTZ) pharmacological model. [20] Furthermore, toxicity estimation by the Lorke method [21] showed that all the aza-Biginelli products studied exhibit a low toxicity profile. It worth noting that, although the results obtained strongly validate the anticonvulsant activity of dihydropyrimidinone JM-II-43A, as already reported by Lewis et al. in their pioneering work, the aza-Biginelli products analyzed here with the best survival rate at a dose of 10 mg/kg turn out to be those compounds that have a strong π donor group at the *para*-position in the phenyl moiety. Overall, the findings suggest a possible relationship between the degree of softness (polarizability) of the aza-Biginelli scaffold and the triggering bioactive protective effect.

## Results and discussion

The design of the aza-Biginelli products, methyl (ethyl)-4-(*p*-arylsubstituted)-6-methyl-3,4-dihydropyrimidin-2(1*H*)-one-5-carboxylate, was focused on the exchange of the hydrogen atom by π donor (OMe, halogen, Me but σ acceptor or σ neutral) substituents at *para*-position of the aryl moiety at position 4 of the 3,4-dihydropyrimidin-2(1*H*)-one scaffold relative to the model JM-II-43A compound. This subtle structural modification, seemingly incapable of inducing significant changes in scaffold, considerably influences the selectivity of the ligand-receptor complex associated with non-covalent interactions, even in the formation of covalent bonds on biological targets. Thus, once the set of aza-Biginelli compounds was synthesized and pharmacologically evaluated, we perform a comparative analysis of the main HSAB parameters between the homologous scaffolds and the model ligand of interest.

**Fig. 2:**
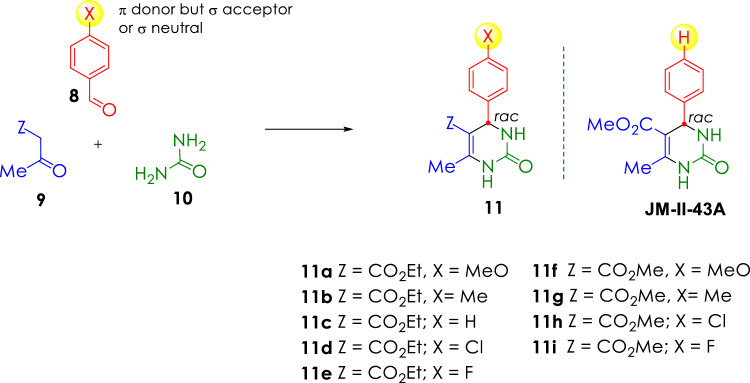
Overview of the multicomponent synthesis of the aza-Biginelli products (**11a**–**i**) and the subtype-selective GABA_A_ modulator JM-II-43A from building blocks **8**–**10**

**Chemistry.** The aza-Biginelli products surveyed pharmacologically in this work were obtained from the same synthetic scheme used for the assembly of the dihydropirimidone model compound JM-II-43A. [22, 23] This multicomponent Biginelli-type reaction leading to the cycloheterocondensation reaction for the non-enantioselective assembly of this privileged scaffold is outlined in Fig. 2. Assuming subtle variations of the electronic effect on the heterocyclic scaffold that should affect its bioactivity profile, resulting from the interaction of the π donor groups in the *para*-position and the π orbitals of the aromatic moiety, a set of aza-Biginelli compounds was then prepared in which, considering the pharmacological effect of the JM-II-43A chemotype, the phenyl moiety and the nucleophilic carbonyl functionality were kept at positions 2 and 4 on the scaffold, while the ethoxycarbonyl or methoxycarbonyl motif was conserved at 5-position in the azaheterocyclic core (**11a** − **i**). Since all their physicochemical and spectroscopic data are available elsewhere, [22, 24–30] we consider it appropriate to dispense with their analysis in this work (See supporting information S3-S12).

### Biological evaluation and HSAB descriptors for adduct formation

The anticonvulsant effect of the subtype-selective GABA_A_ modulator JM-II-43A, along with nine other homologous ligands with different decoration patterns (**11a**–**i**), was evaluated in vivo using the PTZ-pharmacological model. The drug pentylenetetrazol (PTZ) was administered intraperitoneally to 25–30 g male CF1 mice at a dose of 90 mg/kg dissolved in a mixture of DMSO (3.0%) and isotonic saline (0.9%), following administration of 10, 31.6, 100, and 316 mg/kg of the corresponding racemic mixture of test ligands dissolved in an equal volume of the same solvent. The mice were monitored for 30 min to observe the onset of the seizures. Anticonvulsant efficacy was assessed primarily by seizure frequency (Table 1) and the PTZ-induced seizures protection score (Table 2). Survival data are presented in the Supplementary Information (S13).

**Table 1:** Effect of aza-Biginelli products (**11a**–**i**) and JM-II-43A on seizure frequency in the PTZ-induced seizure model

Dose mg/kg	Compounds									
	11a	11b	11c	11d	11e	11f	11g	11h	11i	JM-II-43A
0	11.0 ± 0.41 **a**	11.0 ± 0.41 **a**	11.0 ± 0.41 **a**	11.0 ± 0.41 **a**	11.0 ± 0.41 **a**	11.0 ± 0.41 **a**	11.0 ± 0.41 **a**	11.0 ± 0.41 **a**	11.0 ± 0.41 **a**	11.0 ± 0.41 **a**
10.0	3.1 ± 0.10 **b**	3.0 ± 0.60 **b**	3.0 ± 0.92 **b**	3.0 ± 0.15 **b**	3.0 ± 0.17 **b**	3.0 ± 0.12 **b**	2.0 ± 0.36 **b**	2.0 ± 0.30 **b**	2.0 ± 0.24 **b**	2.0 ± 0.28 **b**
31.6	2.0 ± 0.19 **b**	2.0 ± 0.51 **b**	3.0 ± 0.90 **b**	3.0 ± 0.11 **b**	2.0 ± 0.09 **b**	2.0 ± 0.18 **c**	2.0 ± 0.25 **b**	2.0 ± 0.30 **b**	2.0 ± 0.25 **b**	2.0 ± 0.25 **b**
100.0	3.0 ± 0.10 **b**	3.0 ± 0.53 **b**	3.0 ± 0.97 **b**	3.0 ± 0.12 **b**	2.0 ± 0.12 **b**	3.0 ± 0.11 **c**	3.0 ± 0.12 **b**	2.0 ± 0.31 **b**	2.0 ± 0.35 **b**	2.0 ± 0.28 **b**
316.0	2.0 ± 0.21 **b**	3.0 ± 0.64 **b**	3.0 ± 0.97 **b**	3.0 ± 0.14 **b**	2.0 ± 0.12 **b**	3.0 ± 0.32 **b**	2.0 ± 0.35 **b**	2.0 ± 0.29 **b**	2.0 ± 0.25 **b**	2.0 ± 0.30 **b**

Values represent the mean ± SEM (n = 15). Different letters indicate statistically significant differences (*p* < 0.05, one-way ANOVA).

**Table 2:** Anticonvulsant protection score (P) of the aza-Biginelli (**11a**–**i**) and JM-II-43A products in the PTZ-induced seizure model

Dose mg/kg	f Compounds									
	11a	11b	11c	11d	11e	11f	11g	11h	11i	JM-II-43A
0	0.00 ± 0.00 **a**	0.00 ± 0.00 **a**	0.0 ± 0.00 **a**	0.0 ± 0.00 **a**	0.0 ± 0.00 **a**	0.0 ± 0.00 **a**	0.0 ± 0.00 **a**	0.0 ± 0.00 **a**	0.0 ± 0.00 **a**	0.0 ± 0.00 **a**
10.0	36.00 ± 0.70 **b**	80.00 ± 1.64 **b**	21.20 ± 0.46 **b**	5.50 ± 0.11 **b**	35.00 ± 0.70 **b**	35.00 ± 0.60 **b**	35.00 ± 0.60 **b**	17.50 ± 0.35 **b**	50.00 ± 1.00 **b**	31.50 ± 2.32 **b**
31.6	20.50 ± 0.95 **b**	31.00 ± 0.62 **b**	54.00 ± 1.08 **b**	30.0 ± 0.60 **b**	49.00 ± 0.98 **b**	40.00 ± 0.70 **b**	40.00 ± 0.90 **b**	52.00 ± 1.04 **b**	98.50 ± 1.97 **b**	40.50 ± 1.88 **b**
100.0	22.00 ± 0.44 **b**	51.50 ± 1.03 **b**	40.00 ± 0.80 **b**	22.50 ± 0.45 **b**	83.00 ± 1.74 **b**	36.00 ± 0.66 **b**	39.00 ± 0.78 **b**	84.50 ± 1.69 **b**	80.50 ± 1.61 **b**	105.0 ± 3.12 **c**
316.0	15.00 ± 0.30 **b**	71.00 ± 1.42 **b**	56.50 ± 1.13 **b**	70.50 ± 1.49 **b**	107.0 ± 2.14 **b**	14.00 ± 0.28 **c**	69.00 ± 1.38 **c**	88.00 ± 0.96 **b**	82.50 ± 2.01 **b**	152.0 ± 10.4 **c**

Values represent the mean ± SEM (n = 15). Percent protection (P) was calculated based on the prevention of PTZ-induced seizures. Different letters indicate statistically significant differences (*p* < 0.05, one-way ANOVA).

To validate the in vivo anticonvulsant activity, the bioactivity of the aza-Biginelli compound JM-II-43A from this homologous series was first tested at the concentrations described above. The results support its anticonvulsant potential, as evidenced by changes in mortality rate, seizure frequency, and seizure severity, along with increased protection against PTZ-induced seizures. These findings partially support the implication of GABA_A_ receptor modulation, consistent with the antagonistic behavior previously described by Lewis et al., in their pioneering work.

Unfortunately, this aza-Biginelli ligand showed a feeble protection rate, with survival of 32.25 ± 4.93% at a dose of 10 mg/kg. In contrast, at the highest dose (316 mg/kg) complete protection (100%) *vs* death was achieved, indicating a non-specific and dose-dependent protective effect. Importantly, the results indicate that aza-Biginelli JM-II-43A, together with compounds **11 g**–**i**, markedly reduced seizure frequency by 81.81% (2 *vs* 11 seizures in the PTZ control group), whereas the remaining compounds produced a 72.72% reduction. Furthermore, all tested compounds significantly reduced the incidence of PTZ-induced seizures compared with the control group (*p* < 0.05), as summarized in Table 1. For comparison purposes, the apparent ranking of protective efficacy against mortality was as follows: JM-II-43A (100%) > **11 h** (96.66 ± 2.53%) > **11c** (93.33 ± 1.32%) > **11i** (86.66 ± 3.52%) > **11b** (80 ± 4.19%) > **11e** (74 ± 4.37%) > **11 g** (72 ± 4.3%) > **11d** (53.33 ± 4.37%) > **11a** (19.00 ± 2.94%) > **11f** (6.66 ± 2.50%) (see Supplementary Information S13).

For the sake of comparison, it is worth noting that our findings differ with those previously reported by Matias´s research group, [31] which disclaims the absence of neuroprotective effect (even for molecules with π-donating groups) against seizures induced by the experimental PTZ model in CD1 strain mice weighting 25 to 40 g. In contrast, we herein found that the presence of the π-donor groups (with moderate σ-acceptor character: OMe, Cl) at the *para*-position of the phenyl motif considerably enhances the pharmacological effect. This effect was particularly evident for compounds **11a** and **11 h**, which exhibited higher survival percentages (46.25 ± 2.04% and 46.75 ± 5.20%, respectively) at a dose of 10 mg/kg compared to the model compound JM-II-43A (Supplementary Information S13), as mentioned.

Surprisingly, at higher doses (31.6 mg/kg) of compound **11a**, differences in protection against mortality became minimal (Supplementary Information S13), accompanied by an increasing mortality rate during the second or third seizure (Table 1), and a concomitant decrease in protection score (P) against PTZ-induced seizures. The protection score (P) is an index and may exceed 100 (Table 2). Although seizure severity, as reflected by protection score, was significantly reduced (*p* < 0.05) for all aza-Biginelli derivatives compared to the control group (Table 2), JM-II-43A was the only compound that showed a clear dose-dependent relationship.

The influence of extended π-conjugation in **11a** allows electron delocalization from n_Olp_ orbitals to π_C=C_ phenyl orbitals, which rises the HOMO and LUMO energies, rendering the phenyl unit more suitable as a Lewis base or nucleophile. This overlap modifies their physicochemical parameter, since the n_Olp_ orbitals are good donors and the π_C=C_ orbitals are good acceptors. The strong propensity for canonical forms explains the better polarizability of the phenyl ring, as it become a site with excess π electrons, which transform it into a softer nucleophile substructure, prone to form selective adducts or complexes with partner soft electrophilic receptors, according to the principle of the HSAB model and/or perturbation theory of reactivity. [32, 33] By accounting for this electronic effect, the influence of neutral-hyperconjugation operating on the exocyclic carbonyl unit (${n}_{Nlp}$→${\pi}_{C=O}^{*}$) in heterocyclic ring should not be overlooked, which in this case behaves as a relatively hard site with low polarizability compared to the ${n}_{Nlp}$→${\pi}_{C=S}^{*}$ overlap in monastrol. From this, it could be inferred that the greater the polarizability (softness) effect expressed by the molecule, the greater the biological effect triggered.

Accordingly, the stronger intrinsic σ-acceptor effect induced by the F- atom in the homologous compounds **11e** and **11i**, which results in a less polarizable and comparatively harder ring than compounds **11a** and **11 h** due to the higher Pauling electronegativity of the Fluor atom, in addition to the harder carbonyl site embedded into the scaffold, appears to be consistent with the lower survival rate observed in mice when these molecules were tested, *i.e*., 37.00 ± 5.11 and 33.75 ± 5.19, respectively. On the other hand, although the substitution pattern of the π-acceptor (–CO_2_Me or –CO_2_Et) tethered at C5 (which lower the HOMO and LUMO energy, thus rendering the C5–C6 -moiety less reactive as a Lewis base but more reactive as a Lewis acid) seems to contribute to the observed bioactivity (**11i** vs **d**), the results show a close correlation of bioactivity with the ability of the π-donor substituent to overlap the π-electron system of the aromatic ring (**11a** *vs* **e**). This dependence becomes evident when this substructure presents a weaker π-donor substituent (but σ-neutral), as in case of aza-Biginelli compound **11b**, which showed one of the lowest bioactivities (14.00 ± 1.16) and significant difference when compared with the control group and the additional homologous aza-Biginelli compounds, *p* < 0.05 (except for **11d**). Consequently, the protection factor found at the dose of 10 mg/kg could be ordered as follows: **11a** ≈ **h** > **11f** ≈ **c** > **11e** ≈ **11i** ≈ JM-II-43A ≈ **g** > **11b** ≈ **d**.

On the other hand, since HSAB principles categorize reacting molecules into hard-soft Electrophile–Nucleophile based on frontier molecular orbital (FMO) theory to reliably predict adduct formation from the interaction of their outermost selective orbitals [HOMO (highest occupied molecular orbital)/LUMO (lowest unoccupied molecular orbital)], the FMO energy values associated with the JM-II-43A scaffold as well as its homologous series was obtained from quantum mechanical calculations which were used to compute quantitative HSAB parameters such as softness (σ), hardness (η), and electrophile index (ω) essential to understanding the nature of their reactive sites that could be reasonably correlated with those of their biological partners targets grounded on Pearson´s concepts. Consequently, the results of these quantum mechanical parameters obtained with DF B3LYP/6–311 +  + G** method are collected in Table 3. Among the aza-Biginelli compounds studied, **11a** has been shown to be the relatively strongest nucleophile; as denoted by its computed values of σ and ω, compared to those of other members of the class. This suggests a higher propensity to form stable adducts by donating electrons to an electrophilic site, whether on a protein or a receptor target. Although their propensity to favor other non-covalent complexes is equally important, namely π-π stacking interactions (known to come into play at distances of 3.4–4.4 Å and a dihedral angle of 90°), cannot be ruled out in advance due to their enhanced polarizability associated with the negative value of the quadrupole moment of the phenyl ring. Similarly, its high μ value indicates a highly uneven electron distribution over the molecular framework, giving it properties analogous to those of reactive dipolar species. In this sense, comparison of their respective quantum–mechanical parameters indicates that compounds **11c**, **e**, **i**, and JM-II-43A exhibit comparatively lower nucleophilic character, whereas scaffolds **11b**, **d**, **g**, and **h** display intermediate nucleophilicity. These theoretical findings suggest that the relative differences in nucleophilic behavior are primarily determined by the chemical nature of the substituent groups and their contribution to electron density within the aromatic moiety.

**Table 3:** Calculated quantum mechanical parameters for the model compound JM-II-43A, monastrol, and its homologous aza-Biginelli products **11a**–**i**.*

cmpd	E_**HOMO**_ (eV)	E_**LUMO**_ (eV)	ΔE_(LUMO–HOMO)_^1^ (eV)	IP (eV)	EA (eV)	χ (eV)	η (eV)	σ (× 10^–3^ eV^−1^)	ω (eV)	μ (D)
11a	−6.26	−1.34	4.92	7.754	−0.064	3.845	3.909	255	1.891	3.951
11b	−6.48	−1.43	5.05	8.019	−0.054	3.983	4.037	247	1.965	3.111
11c	−6.48	−1.42	5.06	8.115	−0.074	4.020	4.095	244	1.973	2.859
11d	−6.61	−1.57	5.04	8.163	0.112	4.137	4.025	248	2.126	2.683
11e	−6.59	−1.52	5.07	8.202	0.001	4.102	4.100	243	2.052	2.670
11f	−6.27	−1.37	4.89	7.776	−0.058	3.859	3.917	255	1.901	3.734
11g	−6.45	−1.41	5.04	8.009	−0.097	3.956	4.053	246	1.931	2.885
11h	−6.64	−1.60	5.04	8.192	0.123	4.157	4.034	247	2.142	2.509
11i	−6.62	−1.55	5.07	8.239	0.013	4.126	4.113	243	2.070	2.478
JM-II-43A	−6.51	−1.45	5.06	8.153	−0.076	4.038	4.115	243	1.981	2.652
Monastrol	−6.07	−1.80	4.20	7.664	0.397	4.031	3.634	275	2.235	4.380

*E_LUMO_ and E_HOMO_ for each compound were obtained after calculating ground state equilibrium geometries with the B3LYP/6–311 +  + G** method. These values were generated exclusively from corresponding *s*-*cis* conformations and were used to calculate softness (σ), the electrophilic index (ω) of each electrophile, ionization potential (IP), electron affinities (EA), electronegativity (χ), global hardness (η), and dipole moment (μ). ^1^Energy gap (ΔE) = E_LUMO-_E_HOMO_

The convergence between experimental anticonvulsant response and theoretical softness trends suggests an electronically coherent pattern, rather than a fortuitous association. Although a strict linear quantitative correlation was not the primary goal of this study, the consistent directional agreement between softness trends and biological response reinforces the mechanistic plausibility of the proposed electronic modulation model. For that reason, the better π-donor effect induced by the methoxide group on **11a** (compared to **11c**) significantly contributes to electron density of the phenyl ring, which increases its respective E_HOMO_ while improving its softness (polarizability) character making it good ligand for drug-target interaction. On the contrary, **11e**, bearing a fluorine atom as a strongly σ-accepting substituent, decrease E_HOMO_ and therefore its softness (ΔE_(LUMO–HOMO)_: 4.92 *vs* 5.07 eV), thus expressing a more electrophile character as denoted by the ω value determined by the quantum mechanical method. The results suggest that molecules that develop a stronger σ-acceptor character, given their strong π-conjugative effect and high electronegativity (**11d**–**e**, [$\overline{X }$ = 8.2 eV], **11h**–**i**, $\overline{X }$ = 8.2 eV]) render a higher IP value, stabilized at 0.30 eV (≈ 7 kcal/mol) with respect to those that present weaker σ-acceptor character and improved neutral hyperconjugation (molecules **11a**-**b** [$\overline{X }$= 7.9 eV], **11f**–**g** [$\overline{X }$ = 7.9 eV]). The outcomes indicate that IP is indeed controlled by electronic effects. Overall, a difference of 0.49 eV (≈11.2 kcal/mol) was found between the lowest and highest calculated IP values.

For the sake of comparison, unsubstituted phenyl analogs (**11c** and JM-II-43A), lacking of important electronic perturbations such as π-conjugation or hyperconjugation effect, show slightly lower softness values, with a moderate electrophilic index and intermediate polarity (Table 3). From this point of view, the computed quantum mechanical values suggest the following nucleophilicity order for the examined aza-Biginelli compounds: **11a** = **11f** > **d** > **b** = **11 h** > **g** > **c** > **e** = **11i** = JM-II-43A.

Since FMO theory stand as a powerful tool for predicting chemical reactivity based on the HOMO and LUMO energy level, the computed HOMO–LUMO energy gap values (∆E_Lumo-HOMO_) of the aza-Biginelli products are gathered in Table 3. The ∆E_Lumo-HOMO_ values of all of the compounds ranged from 4.21 to 5.07 eV (~ 97.1–117 kcal/mol). The π-conjugated molecules **11a**, **11f** and monastrol showed narrower ∆E_LUMO-HOMO_ values of 4.92 eV, 4.89 eV and 4.20 eV (~ 113.45, 112.76 and 96.85 kcal/mol, respectively). Although these relatively low energetic values might imply potentially favoured charge-transfer interactions with good redox potential, high polarizability (as could be inferred from the values of the parameters η and σ in Table 3) and low kinetic stability for the facile formation of the activated complex with potential associated biological macromolecules behaving chemically amphoteric system, the fact remains that the obsered energy gaps seems exceptionally very large compared to the limiting gap of 1.30 eV suggested for very reactive compounds. [34] Nevertheless, the lower values of the parameter ω for compound **11a** and **f** indicate a marginal stronger nucleophilic nature than for JM-II-43A compound and monastrol. However, after comparing the electrophilic potency scale described by Domingo-Contreras et al., all the examined aza-Biginelli compounds can be classified as strong electrophiles, since their determined ω values exceed 1.5 eV. [35] Despite the above, it is worth noting that compounds **11d** and **h**, in particular, with relatively lower HOMO and LUMO energy, could behave as Lewis acids and relatively soft electrophiles, being chemically more reactive within the aza-Biginelli series examined. This feature is even more noticeable in the case of monastrol, whose estimated E_LUMO_ was −1.80 eV.

**Fig. 3:**
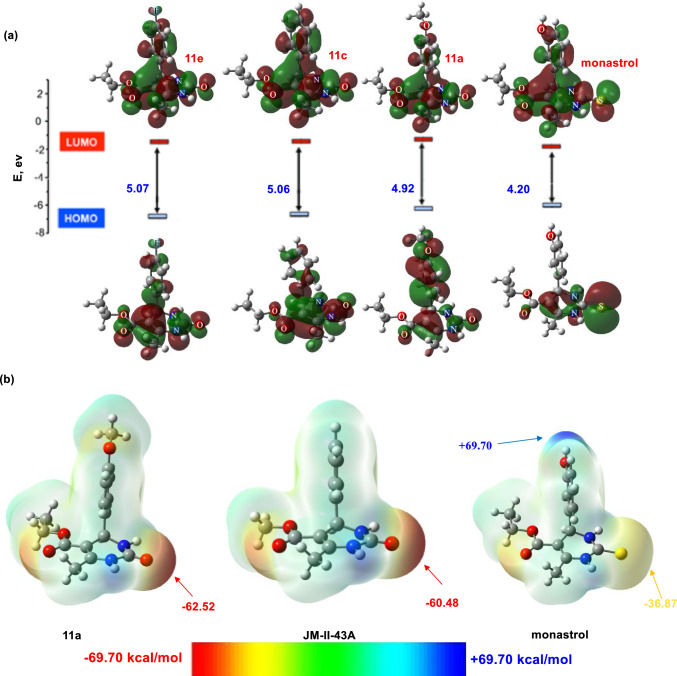
**a** Frontier orbitals of selected molecules (**11a**, **c**, **e** and monastrol) calculated at the B3LYP/6–311 +  + G** level of theory and arranged according to their relative energies. **b** Electrostatic-potential (ESP) surface maps of **11a**, JM-II-43A, and monastrol at the 0.02 e/au^3^ isodensity. The color of the ESP ranges from the high positive potential (blue) to a high negative potential (red)

On the other hand, the isodensity plot of the frontier molecular orbitals and their energy levels computed by B3LYP/6–311 +  + G** method, predicts significant differences in reactivity among all the aza-Biginelli compounds examined (Fig. 3a, top). Highlights that, in the case of monastrol, where the thiocarbonyl motif is located at C2 of the azaheterocyclic ring, the HOMO isodensity was mainly dispersed over the thione group giving rise to a highly polarized molecule (μ = 4.380 D) and leaving the phenyl substructure devoid of density electrons. When it comes to pyrimidinone **11a**, the electron density is essentially distributed in the phenyl ring, unlike to homologues molecules **11e** and **c** that concentrate on the pyrimidine ring, resulting in different dipole moment values (3.951, 2.670 and 2.859 D, respectively). These subtle electronic differences are sufficient to surmise differentiated biological effects, depending on the nature, type and bond strength of the predominant non-covalent molecular interaction, such as donor–acceptor-donor (DAD) interactions sites, Debye forces, π···π stacking interactions. Charge transfer, although to a lesser extent, cannot be ignored.

Accordingly, analysis of the molecular electrostatic-potential (MESP) maps of **11a**, JM-II-43A and monastrol (Fig. 3b, bottom) reveals the latent nature of several key physicochemical parameters intrinsic to these scaffolds, providing insight into the non-covalent interactions essential for drug activity. [36] In this sense, it is evident that the electron density could flows from C = O, CO_2_Et (Me) or S = O function, which remain as the main nucleophile sites, towards the potential partner macromolecules as electrophile. Consequently, the electronegative region of aza-Biginelli adducts is located close to these groups, indicating that an electrophile will be attracted to the negative region, as we see in the MESP plot. These regions, represented in red in the MESP map, exhibit high electron density around the oxygen of the carbonyl, even higher than that of the sulphur of the thionyl in monastrol, in contrast to the electropositive regions of nitrogen atoms that exhibit a positive potential (blue) because of their close location with the C = O group influenced by the hyperconjugative effect (${n}_{Nlp}$→${\pi}_{C=O}^{*}$). Therefore, these regions are prone to form noncovalent bonds, mainly C-H···π stacking and DAD interactions,^8b^ highlighting hydrogen bonding. Meanwhile, electrophilic reactivity is clearly expressed in the C = O or S = O function sites (the red and yellow zones).

**Fig. 4:**
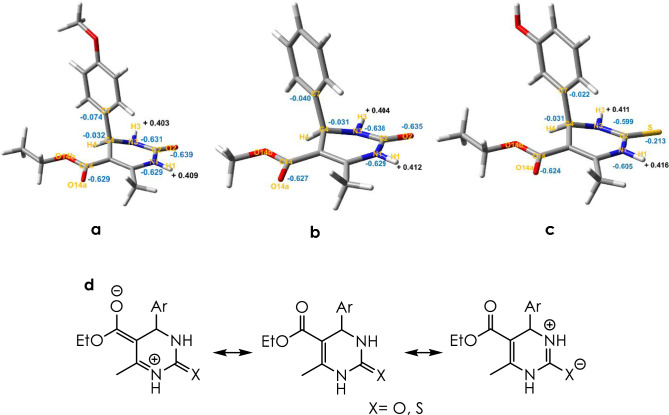
Partial charges obtained from NPA (at B3LYP/6–311 +  + G** levels of theory) are reported as a fraction of the elementary charge (**e**) for **a** the product aza-Biginelli **11a**, **b** JM-II-43A and **c** monastrol, with the atom numbering scheme shown, (for additional information, see supporting information S14-S15). **d** Conjugation between the lone pair of electrons of the endocyclic amino group and the ring π electrons

Matches were found with the analysis of the partial charges obtained from Natural Population Analysis (NPA, Fig. 4a–c) reveals that in compound JM-II-43A, a negative charge of −0.629 is centered on N1, while N3 atom shows a larger negative charge of −0.638, values similar to those of compound **11a** (−0.629 / −0.631) but lower from than those of monastrol (−0.605 / −0.599). The NPA charge values of the C4 and C7 on the phenyl moiety are −0.031/ −0.040 (JM-II43A), −0.032/ −0.074 (11a) and −0.031 /−0.022 (monastrol), respectively. An increase in negative charge is noted at the exocyclic O_2_ atom ( −0.635) tethered to the C_2_ center in comparison to the O14a atom of the carboxylate unit ( −0.627) bonded to the C_14_ center in Compound JM-II-43A. While these values are similar to those found for **11a** ( -0.639 and -0.629, respectively), they differ markedly from those found for the exocyclic sulfur atom bonded to C_2_ in monastrole (−0.213), although subtly for O_14_ (−0.624). From these results, it can be inferred that the binding of a hard-soft electrophile (or Lewis acid) to the hard carbonyl site (either on O_2_ or O_14_b) or to the soft thiocarbonyl site can reduce the electronic charge density on both nitrogen atoms of an aza-Biginelli product. This effect being most pronounced in the monastrol. It worth noting that the hydrogen (or other non-covalent) bonding potential gets improved for the hydrogen bond donor sites. At the same time, the hydrogen bonding potential of the hydrogen bond acceptor sites get improved. In both the cases, these charge redistributions are associated with an enhanced π-conjugation energy of the lone pair of the endocyclic amino group in the aza-heterocyclic system (Fig. 4d) as a consequence of the π-attractor character of these functionalities (which promote a combination of π-extended conjugation and neutral -or negative- hyperconjugation effects).

### Acute toxicity

The lethal dose 50 (LD_50_) of the ten aza-Biginelli products evaluated was determined by the Lorke method. [21] An LD_50_ value greater than 1000 mg/kg of weight was found, which means that none of the tested compounds present significant toxicity.

## Conclusion

The bioactivity and estimation of HSAB parameters were determined as biological predictors of aza-Biginelli adducts structurally related to JM-II-43A-molecule, a selective modulator of the GABA_A_ receptor subtype. Preliminary in vivo anticonvulsant results obtained using the PTZ pharmacological model support that chemical **11a** exhibits improved bioactivity compared to the model compound JM-II-43A at a dose of 10 mg/kg, although less satisfactory outcome was found at higher doses (316 mg/kg). These findings suggest that molecular scaffolds with stronger π-donor substituent with a moderate σ-acceptor character at the phenyl moiety generate the most favorable biological response. Furthermore, the degree of π-conjugation in the exocyclic functionality of the azaheterocyclic skeleton appears to be essential for modulating activity (or biological target selectivity) by enhancing the formation of non-covalent bonding. On the other hand, he quantum-chemical descriptors indicate that the combination of the low polarizability of the exocyclic –NH–C = O in the heterocyclic ring and the higher σ-acceptor capability of the substituent in the phenyl ring gives these electrophilic entities greater kinetic stability.

Although further studies are required, these preliminary outcomes suggest that the aza-Biginelli **11a** product could be considered a good starting point for exploring broader molecular spaces in search of more active aryldihydropyrimidinones prototypes as specific modulator of the GABA_A_ subtype.

## Experimental

### Material and general methods

All drugs, chemical reagents and solvents were obtained from Sigma Chemical Co. (St. Louis, MO, USA) and used as received. The model JM-II-43A compound, as well as the biologically tested aza-Biginelli agents, were prepared and identified by comparing their spectral parameters and physical constants with those reported in the literature.^14 h^

## Biological assays

### Preparation of chemicals and test compounds

Pentylenetetrazole, aza-Biginelli products and the reference compound JM-II-43A, prepared immediately before intraperitoneal administration, were dissolved in DMSO 3% and physiological saline (NaCl 0.9%). All solutions used were freshly prepared.

### *In vivo* anticonvulsant screening

#### Selections of animals

Six to eight-week-old male CF1 mice (25–30 g) from The Institute of Epidemiological Diagnosis and Reference, Mexico City, Mexico, were used throughout this study. Animals were kept and housed under controlled conditioning (22 ± 2 °C, 50–60% relative humidity of and 12 h dark / light cycles). After seven days of acclimatization to laboratory conditions, the animals were randomly assigned to experimental groups of 15 mice each. Food and water were allowed ad libitum during the study period. All tests were performed from 9:00 to 15:00 h to minimize circadian influences on seizure susceptibility. The study was approved by the institutional ethic committee, and all procedures were conducted according to the guidelines for the Use and Care of Laboratory Animals (NOM-062-ZOO-1999, Ministry of Agriculture, México). All animal experiments were carried out in a way that minimizes the number of animals and their sufferings. Each animal was tested once, and was sacrificed 24 h after the end of the observation period under deep ether anesthesia.

### Acute toxicity studies [21]

The median lethal dose 50 (LD_50_) was estimated using the Lorke method. Mice were divided into five groups of three animals each (n = 3 per dose level) and deprived of food for approximately 12 h before the experiment. The compounds JM-II-43A and aza-Biginelli compounds (**11a**–**i**) were administered intraperitoneally at doses of 10, 31.6, 100, 316, and 1000 mg/kg. Animals were observed for 3 h and again at 24 h after administration. Mortality was recorded after 24 h.

### Pentylenetetrazol (PTZ)-induced seizure [37–39]

Five groups (n = 15) of independently housed mice were formed. All groups, except the reference ones were intraperitoneal administered with the corresponding tested compounds in log doses of 10, 31.6, 100, 316 mg/kg. Seizures were induced by a single intraperitoneal injection of PTZ(90 mg/kg), administered 30 min after treatment with the corresponding test compound. Each mouse was monitored for 30 min to record the latency, onset of the first tonic-clonus seizure, and the duration of each episode. Finally, after 24 h, the animals in each group was re-examined to determine survival rate.

The protection (P) exerted by each dose of the anticonvulsant agent tested was calculated as follows:^19b^

$$P= \frac{SL\times TNM}{\sum ({I}_{S} {*D}_{S})}$$

Accordingly, the following parameters were recorded: the total seizure frequency per mouse in each group (Ns, the seizure frequency, total frequency of seizures per mouse during the 30-min observation period), the number of mice with seizures in each group (NMs), the total number of mice in each group (TNM), seizure latency in minutes (SL), seizure duration in minutes (Ds), severity (Is. Score 1: jerking lasting more than 3 s; Score 2: forelimb extension and back arching; Score 3: jump with or without squeal, accompanied by clonus seizures; and Score 4: tonic hind limb extension lasting more than 3 s), and finally the record of survival or the time elapsed until death. Deaths were considered seizures of equal duration to those experienced by the animal at the time of death and to the time elapsed from death to the end of the study period.

## Data analysis

Data are presented as the mean ± SEM (the standard error of the mean). The percentage of dead and convulsed mice, as well as the statistical significance of the difference between the control groups and drug-treated animals was calculated with Fisher’s exact probability test. The latency time, tonic-clonus seizures duration, recovery time, and number of seizure were compared by one-way ANOVA test, combined with the Student Newman-Keuls test. Differences were considered significant when the* p* value was < 0.05 (two-tailed). Statistical tests were performed using Sigma Stat 2.03 (Jandel Corp. SPSS Inc. San Rafael, CA., U.S.A).

### Computational method

All calculations were performed using the GAUSSIAN 09 software. [40] The geometries were optimized using the Becke three-parameter hybrid functional combined with the Lee,

Yang, and Parr correlation [41, 42] (B3LYP) density functional method. The basis set used to fully optimize the selected neutral compounds [43–45] was the 6–311 +  + G**. [46] Vibrational frequencies were also analyzed at the same level of theory. The NPA charges [46] on key atoms was determined. Furthermore, the molecular electrostatic potential (MEP) map [47, 48] for some molecules was examined at the same theoretical level.

**Calculation of HSAB Parameters.** The lowest unoccupied molecular orbital (LUMO) and the highest occupied molecular orbital (HOMO) energies were calculated at the B3LYP/6–311 +  + G** level of theory. On this basis, it was possible to estimate the three major HSAB parameters as follows: global hardness (η) as η = (IP – AE)/2 [49], softness (σ) as the inverse of hardness (1/η), and electrophilicity index (ω) as ω = μ^2^/2η [50].

Other relevant indicators of electronic properties such as electronegativity [χ = (IP + AE)/2], ionization potential [IP = E(N-1) – E(N)], electron affinities [EA = E(N) – E(N + 1)] [51] were also evaluated.

## Supplementary Information

Below is the link to the electronic supplementary material.

Supplementary file1 (PDF 819 kb)

## Data Availability

The data generated and analyzed during this study are included in this published article and its supplementary material. Additional data supporting the findings are available from the corresponding author upon reasonable request.

## Abbreviations

B3LYP: Becke, three-parameter, Lee–Yang–Parr hybrid functional
DAD: Donor–acceptor–donor
DFT: Density functional theory
Ds: Seizure duration (min)
EA: Electron affinity
E_LUMO_: Energy of the lowest unoccupied molecular orbital
E_HOMO_: Energy of the highest occupied molecular orbital
ΔE_(LUMO__–__HOMO)_: Orbital energy gap
FMO: Frontier molecular orbital
GABA_A_: Gamma-aminobutyric acid type-A receptor
HOMO: Highest occupied molecular orbital
HSAB: Hard and soft acids and bases
IP: Ionization potential
Is: Seizure severity index
JM-II-43A: Model aza-Biginelli compound
LUMO: Lowest unoccupied molecular orbital
μ: Dipole moment
NMs: Number of mice with seizures in each group
Ns: Total frequency of seizures in each group
PTZ: Pentylenetetrazole
SEM: Standard error of the mean
SL: Seizure latency (min)
σ: Chemical softness
TNM: Total number of mice in each group
ω: Electrophilicity index
η: Global hardness
χ: Electronegativity
