# Correction: Anticonvulsant activity of aza-Biginelli derivatives related to JM-II-43 A and HSAB-based rationalization of their pharmacological profile

**DOI:** 10.1007/s10822-026-00893-4

**Published:** 2026-07-23

**Authors:** Hulme Ríos-Guerra, Harold Alexis Prada-Ramírez, Benjamín Velasco-Bejarano, Margarita López-Martínez, Judith Espinosa Espinosa-Raya, Alfredo Briones-Arandas, René Miranda-Ruvalcaba, María Inés Nicolás-Vázquez, Raquel Gómez-Pliego

**Affiliations:** 1 Laboratorio de Química Multicomponente, Sección de Química Orgánica, Departamento de Ciencias Químicas, Facultad de Estudios Superiores Cuautitlán, Campo 1, Universidad Nacional Autónoma de México, Av. 1ero de Mayo S/N, Santa María Guadalupe las Torres, C.P. 54740 Cuautitlán Izcalli, Estado de México Mexico; 2 Universidad de los Andes, Bogotá Cundinamarca, Colombia; 3 Laboratorio de Química Medicinal Verde, Sección de Química Orgánica, Departamento de Ciencias Químicas, Facultad de Estudios Superiores Cuautitlán, Campo 1, Universidad Nacional Autónoma de México, Av. 1ero de Mayo S/N, Santa María Guadalupe las Torres, C.P. 54740 Cuautitlán Izcalli, Estado de México Mexico; 4 Departamento de Fisiología y Desarrollo Celular, Instituto Nacional de Perinatología, Montes Urales 800, Col. Lomas de Virreyes, C.P. 11000 Miguel Hidalgo, Ciudad de México, Mexico; 5 Laboratorio Multidisciplinario en Ciencias Biomédicas, Escuela Superior de Medicina, Instituto Politécnico Nacional, Plan de San Luis y Díaz Mirón S/N, C.P. 11340 Miguel Hidalgo, Ciudad de México, Mexico; 6 Laboratorio de Farmacología, Facultad de Medicina Humana, Universidad Autónoma de Chiapas, Décima Sur, esquina con calle Central S/N, C.P. 29000 Tuxtla Gutiérrez, Chiapas Mexico; 7 Laboratorio de Investigación en Química Orgánica-Verde, Sección de Química Orgánica, Departamento de Ciencias Químicas, Facultad de Estudios Superiores Cuautitlán, Campo 1, Universidad Nacional Autónoma de México, Av. 1ero de Mayo s/n, Santa María Guadalupe las Torres, C.P. 54740, Cuautitlán Izcalli, Estado de México Mexico; 8 Laboratorio de Química Computacional, Sección de Química Inorgánica, Departamento de Ciencias Químicas, Facultad de Estudios Superiores Cuautitlán, Campo 1, Universidad Nacional Autónoma de México, Av. 1ero de Mayo S/N, Santa María Guadalupe las Torres, 54740 Cuautitlán Izcalli, Estado de México Mexico; 9 Laboratorio de Microbiología Industrial, Sección de Ciencias de la Salud Humana, Departamento de Ciencias Biológicas, Facultad de Estudios Superiores Cuautitlán, Campo 1, Universidad Nacional Autónoma de México, Av. 1ero de Mayo S/N, Col. Santa María Guadalupe las Torres, C.P. 54740 Cuautitlán Izcalli, Estado de México Mexico

**Correction to: Journal of Computer-Aided Molecular Design** 10.1007/s10822-026-00844-z

In this article, María Inés Nicolás-Vázquez should have been denoted as a corresponding author.

The original article has been corrected.

