# Supplementary material for: Anticonvulsant activity of aza-Biginelli derivatives related to JM-II-43A and HSAB-based rationalization of their pharmacological profile
Source: J Comput Aided Mol Des. 2026 Jun 12;40(1):145. doi: 10.1007/s10822-026-00844-z (PMC13263284; doi:10.1007/s10822-026-00844-z)
Supplement: Supplementary file 1 — Supplementary file1 (PDF 819 kb) [file 10822_2026_844_MOESM1_ESM.pdf]

Anticonvulsant activity of aza-Biginelli derivatives related to JM-II-43A and HSAB-based rationalization of their pharmacological profile

Hulme Ríos-Guerra[a], Harold Alexis Prada-Ramírez[b], Benjamín Velasco Bejarano[c], Margarita López-Martínez[d], Judith Espinosa Raya[e], Alfredo Briones-Arandas[f], René Miranda-Ruvalcaba[g], María Inés Nicolás-Vázquez[h] *, Raquel Gómez-Pliego[i] *.

[a]Laboratorio de Química Multicomponente, [c]Laboratorio de Química Medicinal Verde, [g]Laboratorio de Investigación en Química Orgánica-Verde, [h]Laboratorio de Química Computacional, Sección de Química Orgánica, Departamento de Ciencias Químicas, [i]Laboratorio de Microbiología Industrial, Sección de Ciencias de la Salud Humana, Depto. de Cs Biol., FESC1-UNAM, Av. 1[ero] de Mayo S/N, Col. Sta. Ma. Gpe las Torres, Cuautitlán Izcalli, C.P. 54743, EdoMex, México.

[b]Universidad de los Andes, Bogotá Cundinamarca, Colombia.

[d]Departamento de Fisiología y Desarrollo Celular, Instituto Nacional de Perinatología. Calzada Montes Urales 800, Lomas de Virreyes, Lomas de Chapultepec IV Sección, Miguel Hidalgo, C.P. 11000. Ciudad de México, México.

[e]Laboratorio Multidisciplinario en Ciencias Biomédicas, Escuela Superior de Medicina, Instituto Politécnico Nacional, Plan de San Luis y Díaz Mirón S/N, Miguel Hidalgo, C.P. 11340, Ciudad de México, México.

[f]Laboratorio de Farmacología, Facultad de Medicina Humana. Universidad Autónoma de Chiapas. Décima Sur, esquina con calle Central S/N, Tuxtla Gutiérrez, Chiapas, México, C.P. 29000.

Corresponding author: Raquel Gómez-Pliego, pliegoraquel@comunidad.unam.mx, María Inés Nicolás-Vázquez, nicovain@comunidad.unam.mx.

Table of Contents

Materials and General methods	S3
^{1}H NMR spectra for **DHPM** (**11a**-**i**)	S4-S11
^{1}H NMR spectra for compound **JM-II-43A**	S12
Survival percentage	S13
Partial charges obtained from NPA	S14-S15

Materials and General methods

All reagents are commercial products acquired from Sigma-Aldrich and were used as received without further purification. Analytical thin-layer chromatography (TLC)were performed on precoated Kieselgel 60F254 plates, with spot detection under UV light (254 nm). Melting points were determined using a Fisher-Johns apparatus (*reported). Proton nuclear magnetic resonance (^{1}H-NMR) spectra were recorded using a 300 MHz Varian Gemini spectrometer in DMSO-d6 as the solvent and TMS as internal reference.

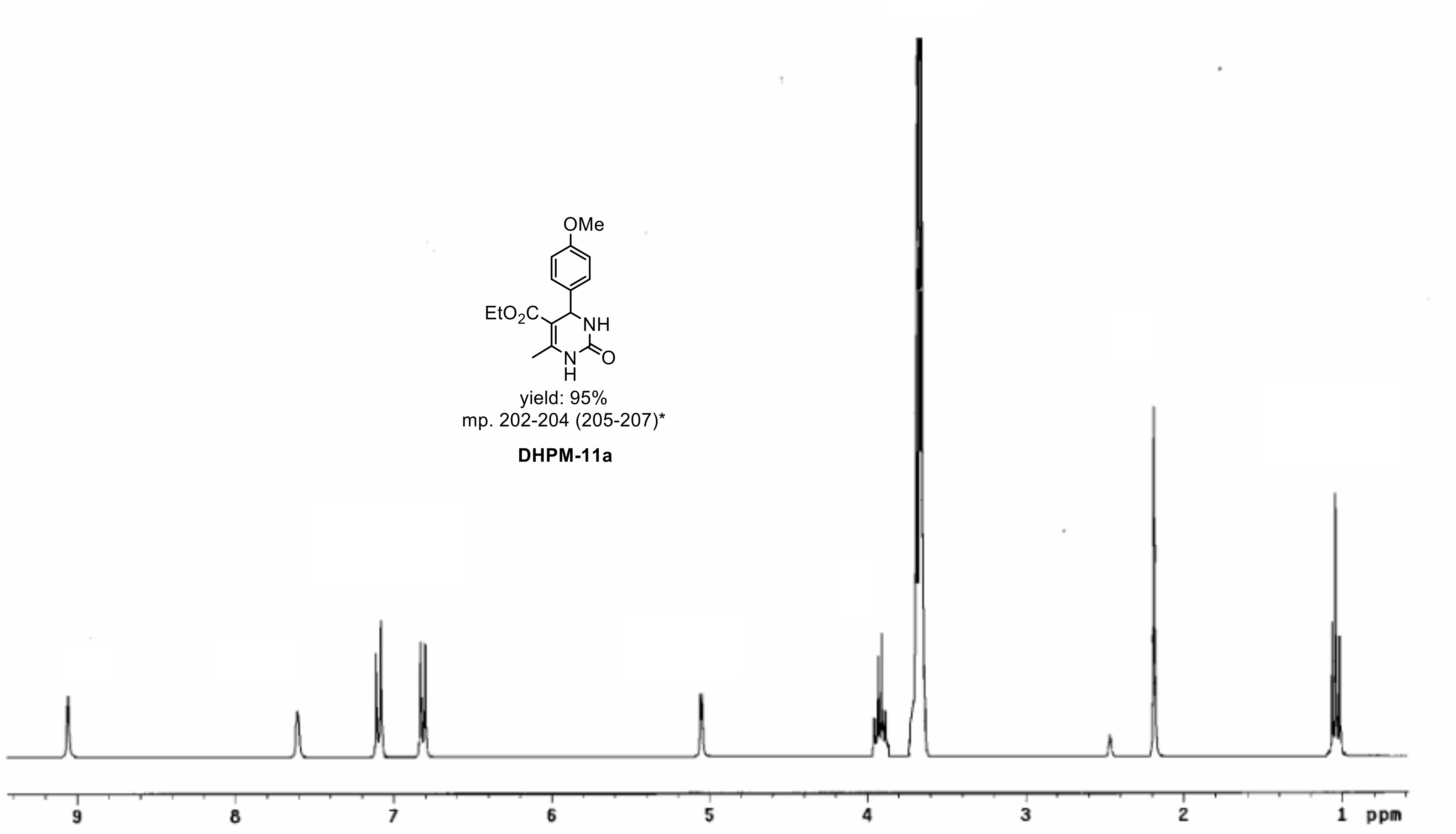
OMe
EtO2C
NH
N
H
O
yield: 95%
mp. 202-204 (205-207)*
DHPM-11a
9
8
7
6
5
4
3
2
1 ppm

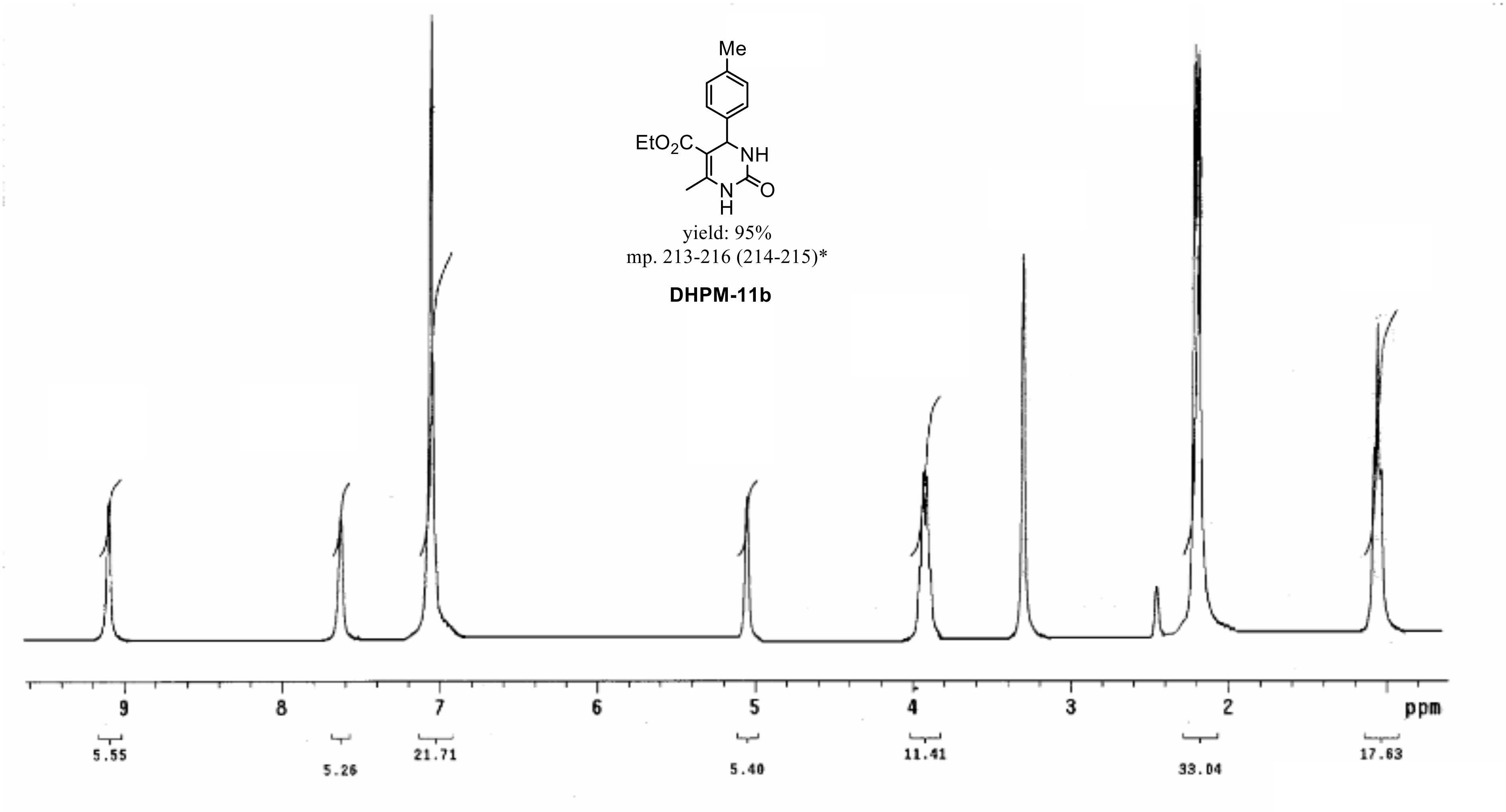
Me
EtO2C
NH
O
N
H
yield: 95%
mp. 213-216 (214-215)*
DHPM-11b
9
8
7
6
5
4
3
2
ppm
5.55
5.26
21.71
5.40
11.41
33.04
17.63

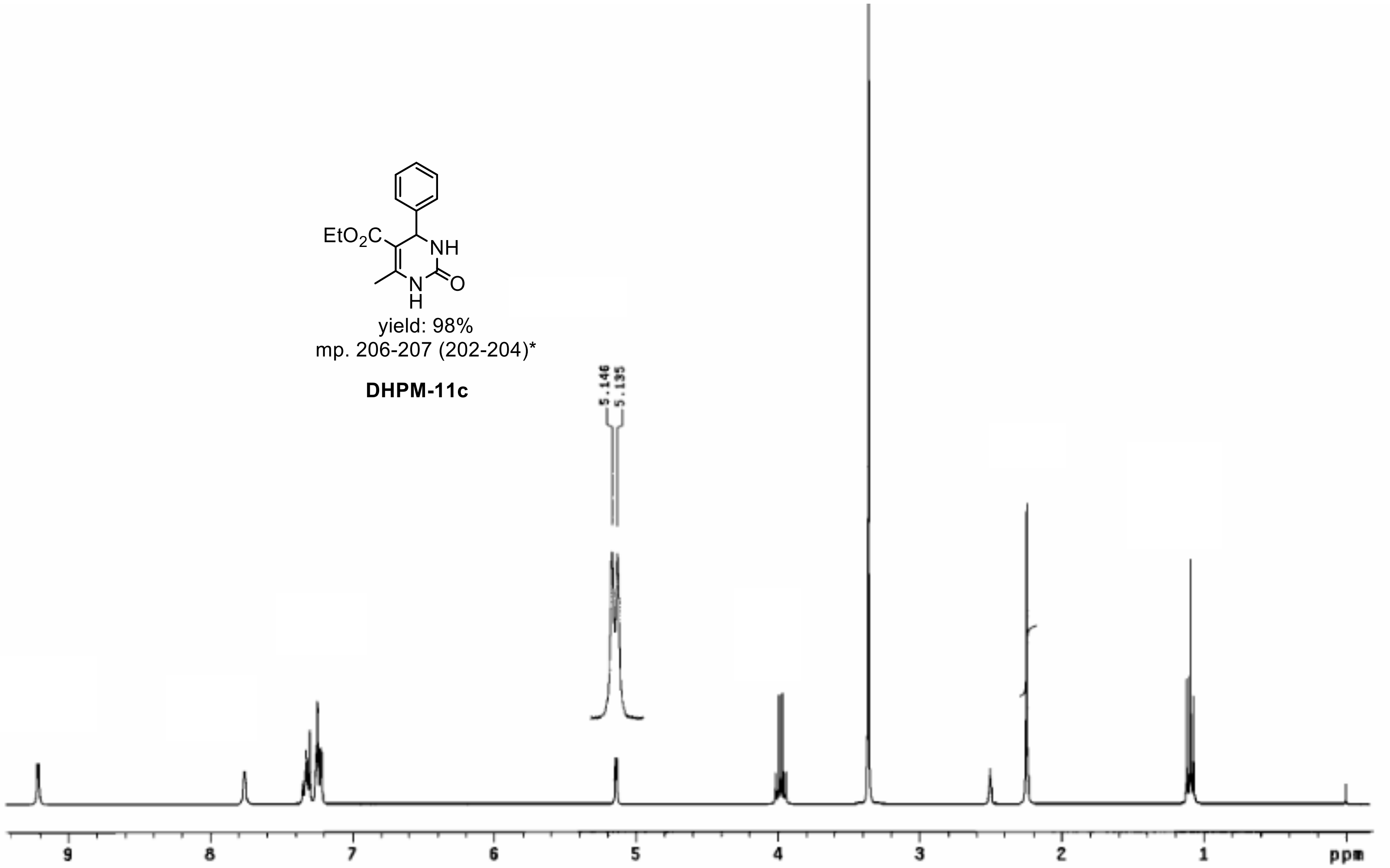

yield: 98%
mp. 206-207 (202-204)*

DHPM-11c

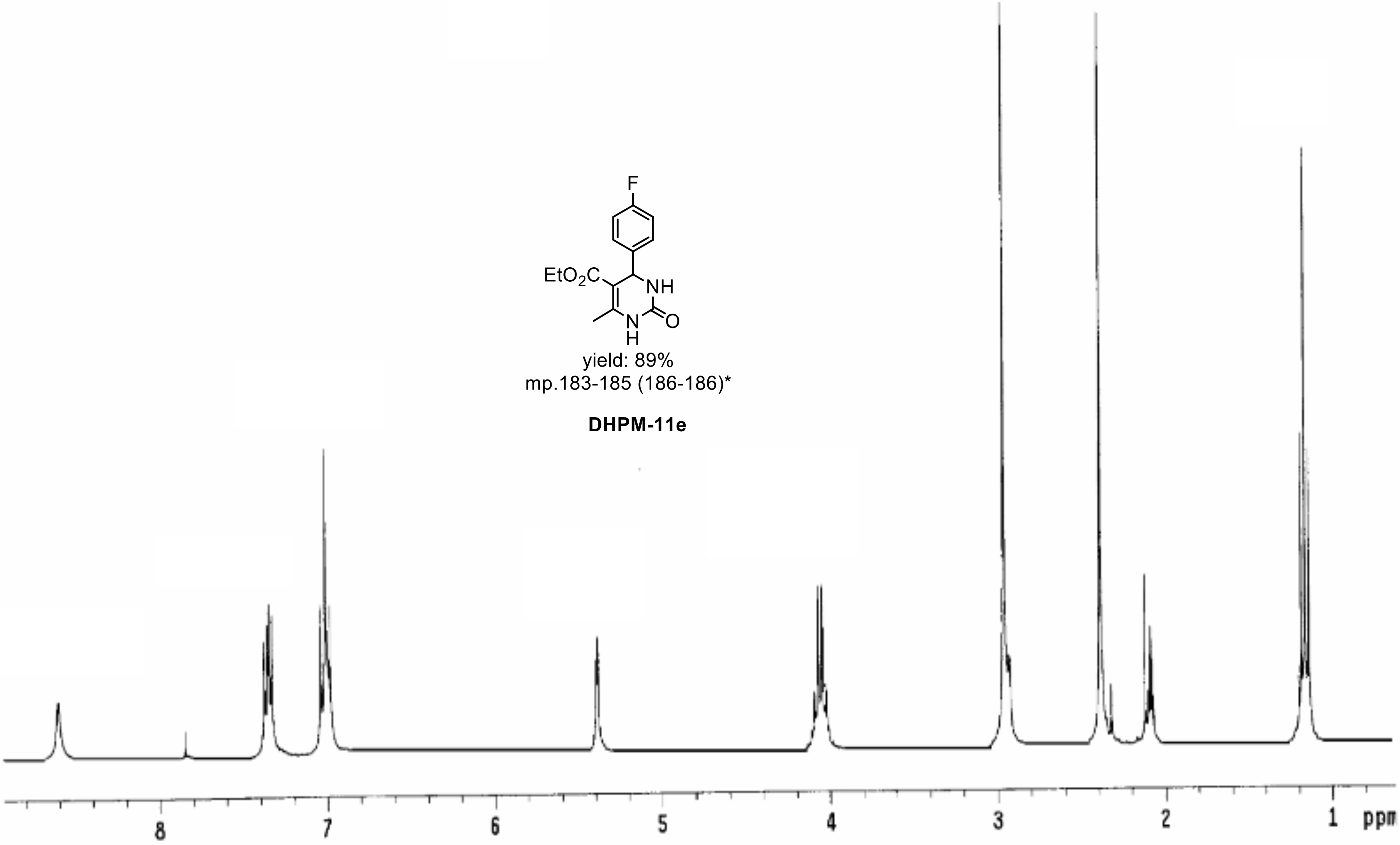
F
EtO2C
NH
N
H
O
yield: 89%
mp.183-185 (186-186)*
DHPM-11e
8
7
6
5
4
3
2
1
ppm

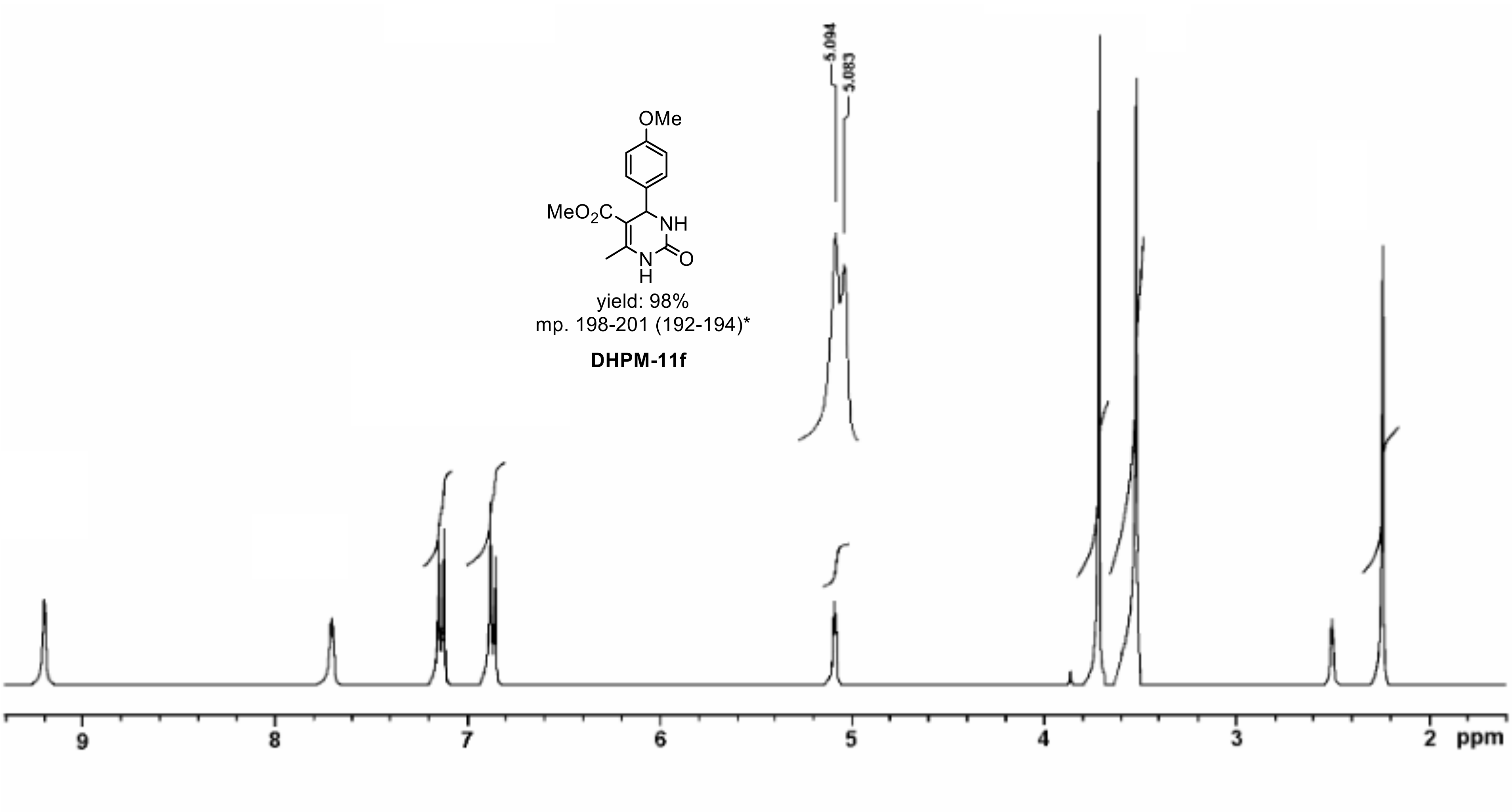
5.094
5.083
OMe
MeO2C
NH
N
H
O
yield: 98%
mp. 198-201 (192-194)*
DHPM-11f
9
8
7
6
5
4
3
2
ppm

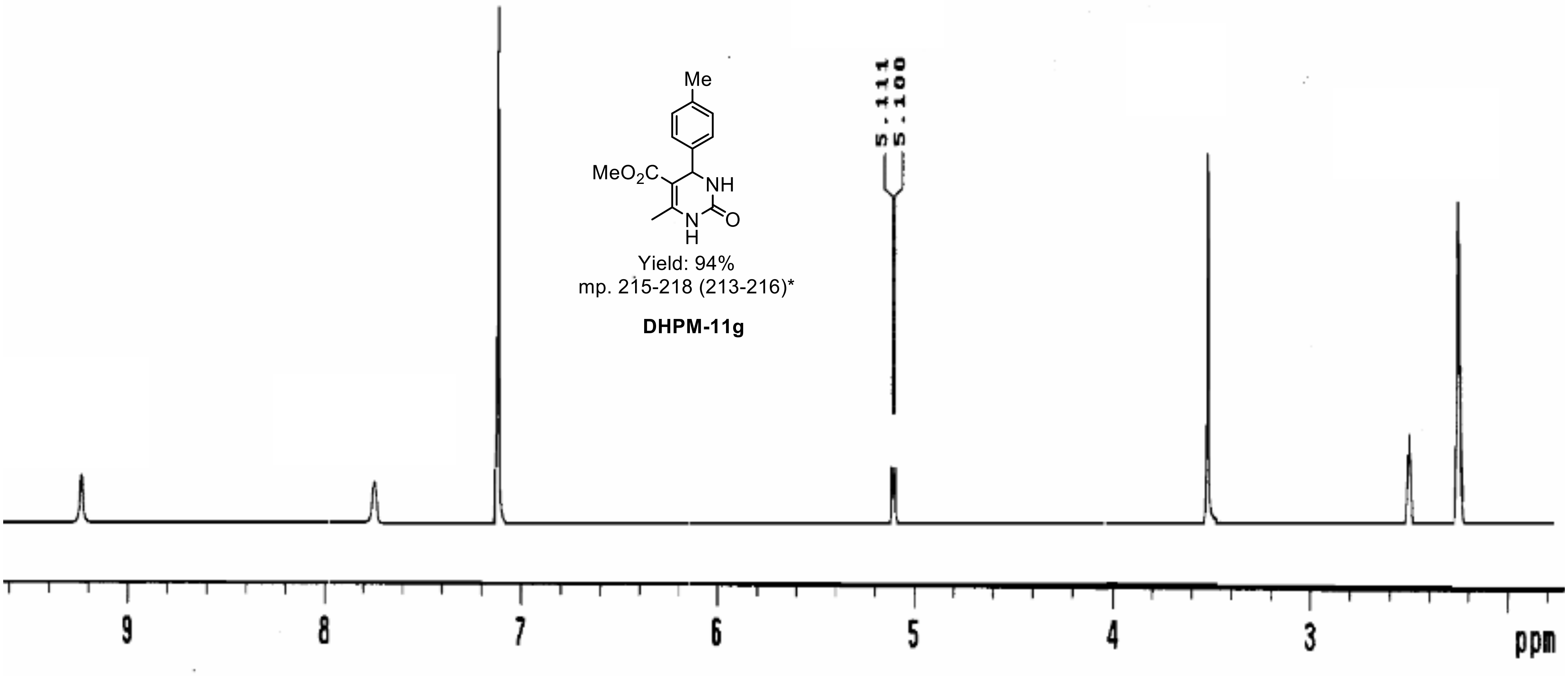
Me
MeO_2C
NH
O
N
H
Yield: 94%
mp. 215-218 (213-216)*
DHPM-11g
5.111
5.100
9
8
7
6
5
4
3
ppm

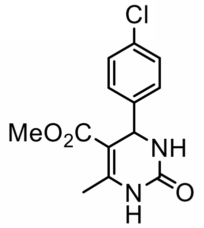

Yield: 90%
mp. 208-210 (204-207)*

DHPM-11h

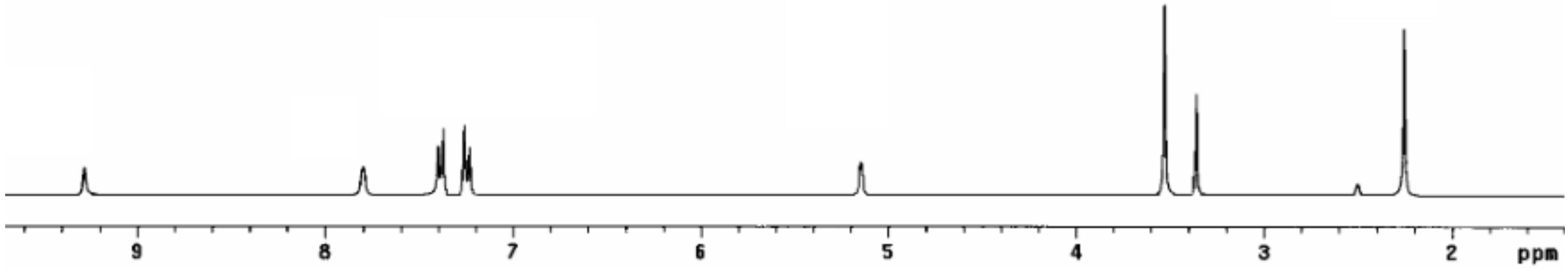

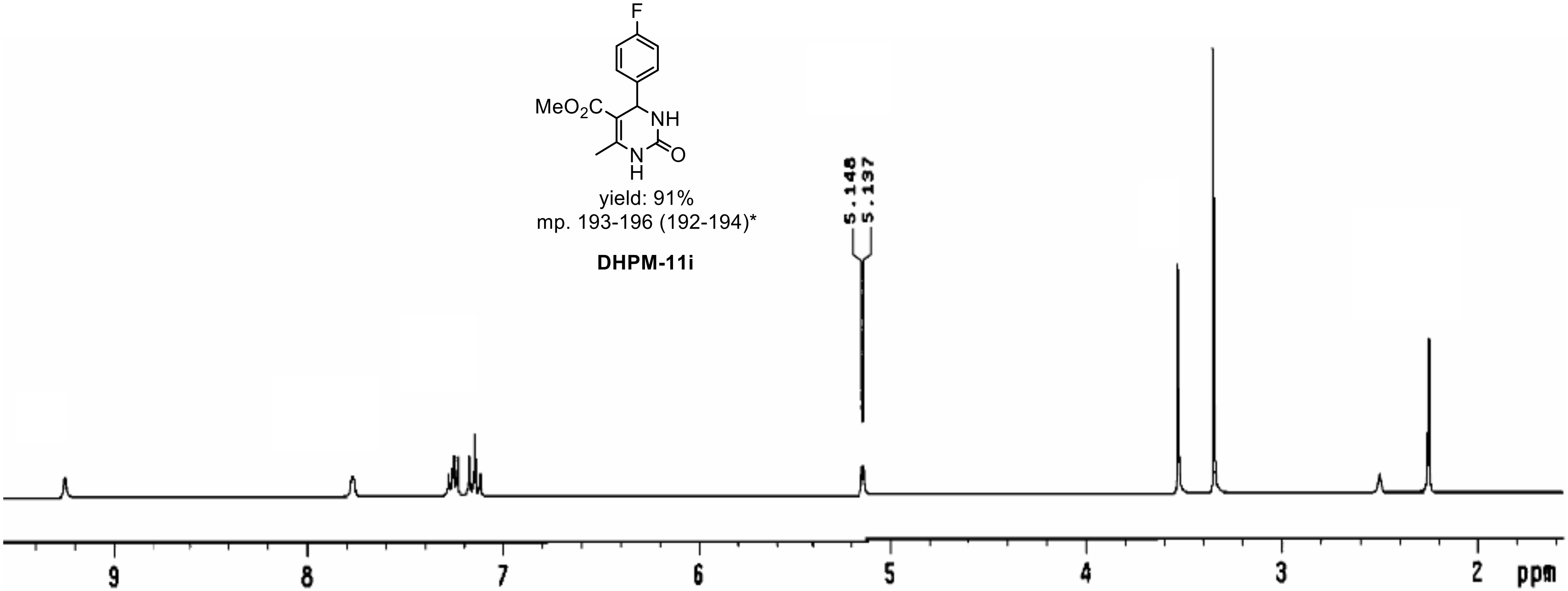

yield: 91%
mp. 193-196 (192-194)*

DHPM-11i

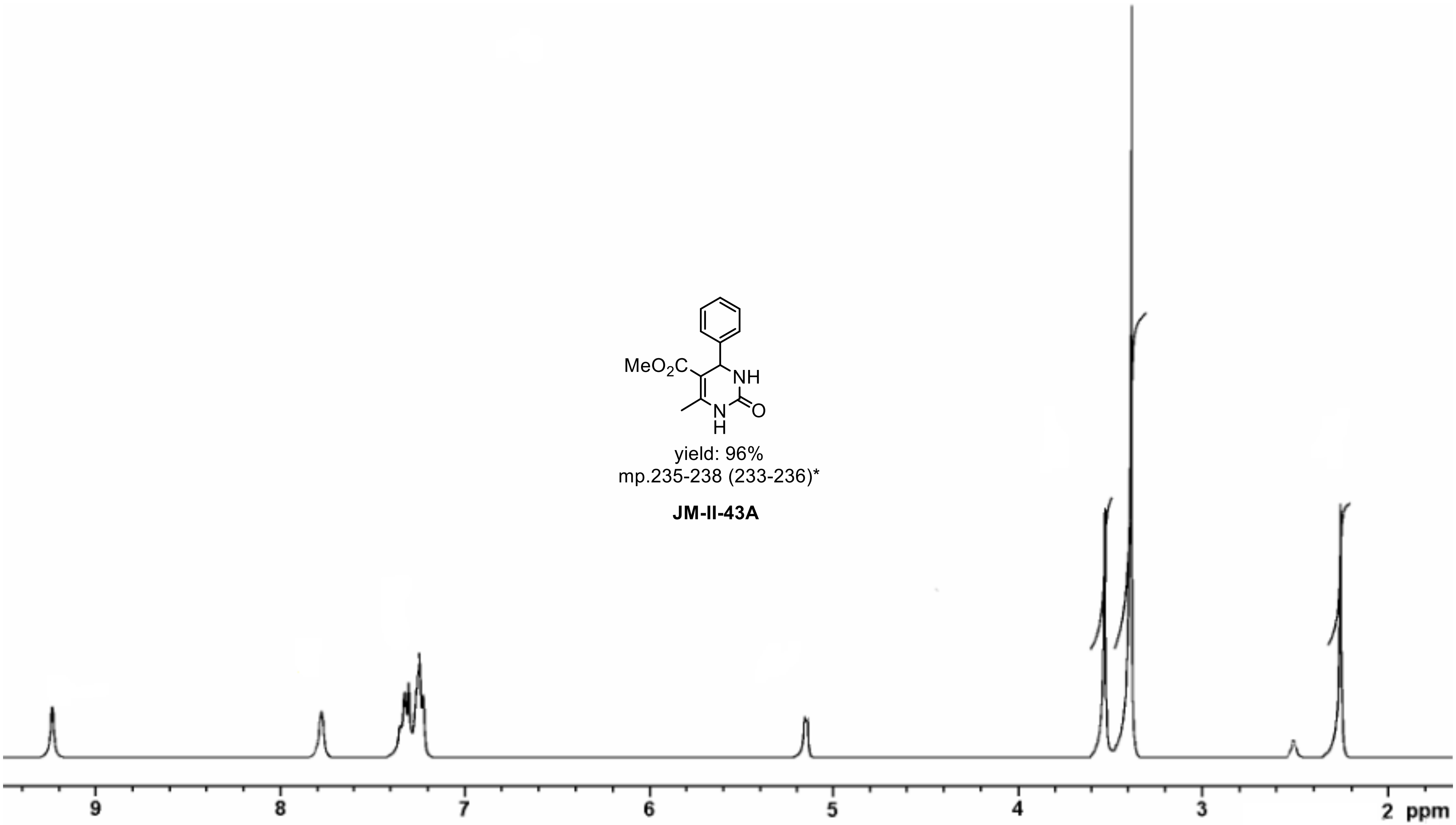
MeO2C
NH
N
H
O
yield: 96%
mp.235-238 (233-236)*
JM-II-43A
9
8
7
6
5
4
3
2 ppm

Table 1. Effect of aza-Biginelli products on the survival percentage.

dose mg/Kg / cmpds	11a	11b	11c	11d	11e	11f	11g	11h	11i	JM-II-43A
**0**	6.00±2.53<br>**A**	6.75±2.6<br>**a**	6.75±4.63<br>**a**	6.75±2.87<br>**a**	6.75±2.87<br>**a**	6.75±2.49<br>**a**	6.50±2.62<br>**a**	6.75±2.00<br>**a**	6.75±2.60<br>**a**	6.75±2.62<br>**a**
**10.0**	46.25±2.04<br>**B**	14.00±1.16<br>**a**	41.75±2.33<br>**b**	11.75±5.11<br>**a**	37.00±5.11<br>**b**	42.25±4.88<br>**b**	30.75±4.65<br>**b**	46.75±5.20<br>**b**	33.75±5.09<br>**b**	32.5±4.93<br>**b**
**31.6**	46.00±2.81<br>**b**	20.50±5.21<br>**a**	54.00±2.67<br>**b**	11.75±4.67<br>**a**	65.50±4.67<br>**b**	23.00±5.18<br>**a**	39.25±5.14<br>**b**	60.00±5.10<br>**b**	47.00±5.19<br>**b**	36.75±5.16<br>**b**
**100.0**	30.50±2.50<br>**b**	33.75±4.9<br>**b**	70.0±2.12<br>**b**	31.50±4.64<br>**b**	65.50±4.6<br>**b**	16.50±3.40<br>**a**	46.00±5.24<br>**b**	87.75±0.95<br>**b**	80.25±3.52<br>**b**	86.5±5.59<br>**b**
**316.0**	19.00±2.94<br>**a**	80.00±4.19<br>**b**	93.33±1.32<br>**b**	53.33±4.37<br>**b**	74.00±4.37<br>**b**	6.66±2.50<br>**a**	72.00±4.30<br>**b**	96.66±0.95<br>**b**	86.66±3.52<br>**b**	100.0±0.00<br>**b**

Effect of aza-Biginelli products on the survival percentage: each point represents the mean of n = 15 ± SEM. Literal differences $p<0.05$. Fisher´s exact test.

Table 2. Partial charges obtained from NPA (at B3LYP/6–311++G** levels of theory) are reported as a fraction of elementary charge (e) for JM-II-43A, monastrol, and aza-Biginelli products **11a**-**11i**, with the numbering of the skeleton atoms indicated.

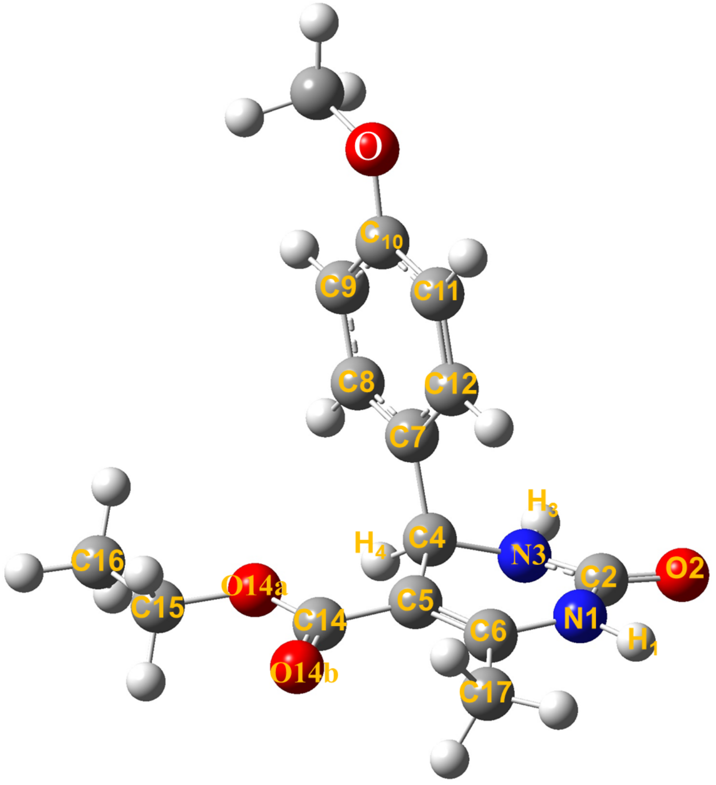

cmpd	11a	11b	11c	11d	11e	11f	11g	11h	11i	JM-II-43A	*Monastrol*
**N_1**	-0.629	-0.636	-0.629	-0.629	-0.630	-0.629	-0.630	-0.630	-0.630	-0.629	-0.605
**H_1**	0.409	0.408	0.409	0.410	0.410	0.409	0.411	0.412	0.412	0.412	0.416
**C_2**	0.811	0.815	0.814	0.814	0.813	0.811	0.813	0.813	0.813	0.814	0.288
**O_2/S**	-0.639	-0.638	-0.636	-0.633	-0.634	-0.638	-0.637	-0.632	-0.633	-0.635	-0.213
**N_3**	-0.631	-0.638	-0.638	-0.638	-0.638	-0.631	-0.640	-0.641	-0.641	-0.638	-0.599
**H_3**	0.403	0.403	0.403	0.404	0.404	0.403	0.403	0.404	0.404	0.404	0.411
**C_4**	-0.032	-0.037	-0.034	-0.035	-0.035	-0.032	-0.021	-0.021	-0.021	-0.031	-0.031
**H_4**	0.222	0.224	0.222	0.223	0.223	0.222	0.222	0.223	0.222	0.222	0.226
**C_5**	-0.230	-0.221	-0.230	-0.228	-0.228	-0.230	-0.233	-0.235	-0.235	-0.231	-0.225
**C_6**	0.283	0.282	0.285	0.284	0.283	0.284	0.285	0.286	0.286	0.286	0.280
**C_7**	-0.074	-0.054	-0.044	-0.046	-0.060	-0.075	-0.054	-0.047	-0.060	-0.040	-0.022
**C_8**	-0.173	-0.188	-0.197	-0.179	-0.176	-0.173	-0.188	-0.178	-0.176	-0.197	-0.244
**C_9**	-0.288	-0.198	-0.197	-0.219	-0.261	-0.287	-0.198	-0.219	-0.260	-0.196	0.322
**C_{10}**	0.326	-0.027	-0.200	-0.035	0.413	0.326	-0.027	-0.035	0.413	-0.201	-0.280
**C_{11}**	-0.229	-0.197	-0.198	-0.219	-0.260	-0.229	-0.192	-0.215	-0.256	-0.193	-0.171
**C_{12}**	-0.176	-0.177	-0.185	-0.168	-0.165	-0.176	-0.186	-0.178	-0.075	-0.195	-0.225
**C_{14}**	0.790	0.796	0.790	0.790	0.790	0.787	0.788	0.787	0.787	0.787	0.790
**O_{14a}**	-0.629	0.630	-0.628	-0.626	-0.627	-0.627	-0.627	-0.624	-0.625	-0.627	-0.624
**O_{14b}**	-0.575	-0.573	-0.573	-0.575	-0.575	-0.562	-0.562	-0.564	-0.563	-0.561	-0.571
